# Toward viewing behavior for aerial scene categorization

**DOI:** 10.1186/s41235-024-00541-1

**Published:** 2024-03-26

**Authors:** Chenxi Jiang, Zhenzhong Chen, Jeremy M. Wolfe

**Affiliations:** 1 School of Remote Sensing and Information Engineering, Wuhan University, Wuhan, Hubei China; 2 Hubei Luojia Laboratory, Wuhan, Hubei China; 3 Harvard Medical School, Boston, MA USA; 4 Brigham & Women’s Hospital, Boston, MA USA

**Keywords:** Aerial image viewing, Scene categorization, Eye movements, Image statistics

## Abstract

Previous work has demonstrated similarities and differences between aerial and terrestrial image viewing. Aerial scene categorization, a pivotal visual processing task for gathering geoinformation, heavily depends on rotation-invariant information. Aerial image-centered research has revealed effects of low-level features on performance of various aerial image interpretation tasks. However, there are fewer studies of viewing behavior for aerial scene categorization and of higher-level factors that might influence that categorization. In this paper, experienced subjects’ eye movements were recorded while they were asked to categorize aerial scenes. A typical viewing center bias was observed. Eye movement patterns varied among categories. We explored the relationship of nine image statistics to observers’ eye movements. Results showed that if the images were less homogeneous, and/or if they contained fewer or no salient diagnostic objects, viewing behavior became more exploratory. Higher- and object-level image statistics were predictive at both the image and scene category levels. Scanpaths were generally organized and small differences in scanpath randomness could be roughly captured by critical object saliency. Participants tended to fixate on critical objects. Image statistics included in this study showed rotational invariance. The results supported our hypothesis that the availability of diagnostic objects strongly influences eye movements in this task. In addition, this study provides supporting evidence for Loschky et al.’s (Journal of Vision, 15(6), 11, 2015) speculation that aerial scenes are categorized on the basis of image parts and individual objects. The findings were discussed in relation to theories of scene perception and their implications for automation development.

**Supplementary Information:**

The online version contains supplementary material available at 10.1186/s41235-024-00541-1.

## Introduction

Living on the ground, we are naturally drawn to explore our surroundings, observing and adapting to the arrangement of the world in a gravitational frame. Daily experience tunes us to be adept at processing terrestrial images, which refer to views available from a viewpoint situated on or near the Earth's surface, typically at eye level. Early pioneers envisioned transcending the grip of gravity to witness the world from a God's-eye view, which became a reality with the advent of aerial photography. In 1858, French photographer and balloonist Gaspar Felix Tournachon, also known as "Nadar," produced the first known examples of aerial photography (Cosgrove & Fox, 2010).

“Aerial photography” pertains to visual data captured from elevated viewpoints, such as from an airborne platform or satellite. These images offer a top-down view of the landscape. Such images may vary in imaging angles, such as oblique satellite photogrammetry. The present work focused on vertical aerial images, derived from Google Earth (Loschky et al., 2015; Xia et al., 2017), a platform providing ortho-rectified aerial imagery.

Nowadays, with the advancement of both photography and space technology, the number and resolution of aerial images have markedly increased with at least one basic goal unchanged: procuring geospatial information on the ground from above. One typical type of this information is the scene category. High-resolution aerial images covering a large area of a city enable users to identify land uses, land covers, and other scene properties of different regions in the city without extensive and laborious site investigation. Indeed, after extracting geoinformation using aerial images, onsite visits serve more as a method to supplement and verify the aerial information (Jiang et al., 2022; Zhao et al., 2018).

In addition to manual interpretation, bio-inspired computational intelligence methods (e.g., convolutional neural networks and vision transformer) have markedly boosted machine performances in remote sensing image (RSI) processing (Aleissaee et al., 2023; Zhong et al., 2018). These bio-inspired methods have the merits of considerable cross-modal and cross-domain generalizability. However, model evaluation and optimization for aerial visual tasks have been primarily grounded in theories of human visual attention as understood from terrestrial viewpoints. For example, humans prioritize shape (95.9%) over texture when categorizing objects in natural everyday scenes (Geirhos et al., 2018). Based on this revelation, in RSI processing, a model is considered better aligned with human visual perception when it shows a stronger shape-over-texture bias (Dehghani et al., 2023). However, whether the same bias applies to humans’ aerial image processing is not clear. In fact, it is well known that both human and computer interpretations of RSIs rely on texture analysis. To enhance RSI-oriented algorithm innovation and move toward full automation, it is crucial to understand how humans process aerial images.

Since none of us have lived in the sky, a core question is our ability to identify landscape features and infer the practical use of unfamiliar areas in aerial images. Interestingly, this process appears to be intuitive for us, at least to some extent. For instance, we can roughly imagine the bird’s-eye view of our living areas without direct observations from above. Indeed, geographers can categorize objects in aerial images into land use with good accuracy even if they do not have extensive experience with either the categorization processes or with aerial photographs (Lloyd et al., 2002). Moreover, observers with little geographic knowledge also categorize aerial scenes with well above-chance accuracy, after processing those images as shortly as 24 ms (Loschky et al., 2015, Experiment 1).

This ability to generalize ground-based knowledge to an aerial viewpoint is believed to be closely related to the hierarchical information processing of visual input in both avian (for a review, see Pusch et al., 2023) and mammalian (Riesenhuber & Poggio, 2000; Vinken et al., 2016) sensory systems. Neuron groups at higher stages of the processing hierarchy in mammals (e.g., primate inferior temporal cortex) exhibit selectivity for complex shapes and invariance to nonlinear changes to some degree such as viewpoint (Bao et al., 2020; Freiwald & Tsao, 2010). Thus, when we roam around our neighborhood, we are likely to be unconsciously creating a mental map of our surroundings, including our little house models as well as the park, nearby supermarket, etc.

This hierarchical mechanism of extracting viewpoint-invariant information and the aerial viewpoint have sparked interest in comparing aerial and terrestrial scene categorization. Some aspects of behavior are similar. For instance, in Pannasch et al.’s (2014) study, they found for both types of images, observers’ fixation durations kept increasing and saccade amplitudes kept decreasing from beginning to end of viewing, with eye movement data binned in 2-s bins. On the other hand, the distinctive characteristics of aerial images become evident when images are rotated. While accuracy in categorizing terrestrial images was significantly reduced upon rotation, performance was stable for aerial images across different image orientations (Loschky et al., 2015, Experiment 2), leading researchers to conclude that the useful information for aerial scene categorization was likely to be orientation-/rotation-invariant. A following experiment justified this notion by showing that texture, a rotation-invariant feature, contributed to rapid scene categorization in aerial condition but not in terrestrial condition (Loschky et al., 2015, Experiment 3). Given that aerial categorization accuracy was approximately 20% when only texture information was available, which was about one-third of the accuracy achieved with intact images, there must be other features that also provide categorical knowledge and affect aerial image processing.

Some studies have exclusively focused on human cognition of aerial images. Certainly, aerial search depends on low-level features such as target size and location, and search template clarity (Rhodes et al., 2021). Rhodes et al. (2021) also suggested that search performance levels off around ten found targets when there are more than ten target instances in the image. This might be true; however, it could be also attributed to their experimental design, with the majority of trials (97%) having one to ten targets. In fact, when foraging for an unknown number of gas stations in satellite images, observers chose to proceed to the next, new image when the expected rate of target collection in the current image fell to an average rate of the environment, a rate-optimizing foraging strategy (Ehinger & Wolfe, 2016; Oaten, 1977). Lloyd and Hodgson (2002) identified serial searches for target-present trials and parallel searches for target-absent trials in aerial images. Specifically, when determining the absence or presence of a target object in black and white aerial images, the increase in the number of objects (e.g., cemetery) associated with the corresponding primary objects (e.g., church) in scenes shortened response times only for target-present trials, and the decrease in the distance between these objects contributed to faster responses for both target-absent and -present trials. In addition, expertise also matters. Sophisticated interpretation of aerial images requires extensive experience/learning (Lloyd et al., 2002). Compared to untrained observers, experienced participants were adept at leveraging semantic information in such images to perform tasks like change detection and delayed memory retrieval (Lansdale et al., 2010; Šikl et al., 2019).

While these studies have separately examined the impacts of different image features on different visual tasks, there is still relatively little known about how people recognize aerial scenes. Which kinds of features are used? How are attention and the eyes deployed during categorization. A better understanding of these aspects of human aerial scene perception could be useful when human and machine talents are combined in RSI tasks. For instance, when a task primarily depends on high-level features, users might personalize image compression protocols to better preserve useful features and reduce the negative impact of compression-related image degradation on task performance (e.g., Xiang et al., 2023).

### The current study

In this study, we explored viewing behavior for aerial scene categorization and factors that influence the eye movements in this process. Given that aerial image processing is often a task for experts, we focused on the viewing behavior of experienced observers. The eye movement metrics used include fixation duration, the number of fixations, saccade amplitude, the entropy of fixation density map, and gaze transition entropy (scanpath randomness).

Computational image analysis methods were used to calculate various image features and statistics at low, mid, and high levels. To extract low-level feature, we employed a set of Gabor filters due to its ability to simulate the response of the early visual cortex to natural images (Henderson et al., 2023; Kay et al., 2008; Lescroart & Gallant, 2019). Low-level image statistic was defined as the mean of Gabor filter responses. For mid-level feature, we used Gray-Level Cooccurrence Matrix (GLCM), a widely accepted texture analysis method in remote sensing and medical images (Alvarenga et al., 2007; Lane et al., 2014). Homogeneity, a second-order measure based on the GLCM was our specific mid-level image statistic. High-level image features are typically considered to include higher-order visual and/or semantic information. Deep features of neural network serve as a surprisingly effective metric to measure perceptual similarity between images (Zhang et al., 2018). Therefore, deep features were used as our high-level feature. Perceptual similarity across aerial images based on image deep features was used as high-level image statistics for both within- and across-category conditions. To expand the range of image statistics and reduce potential side effects of non-exhaustive predictors in regression analyses, we also used outputs from other layers of the neural network model.

We hypothesized that the availability of critical objects influences observers’ eye movement patterns during aerial scene categorization. Some natural scenes may contain key objects or regions that, in Yarbus’ words, “in the observer’s opinion, may contain, information useful or essential for perception” (Yarbus, 1967, pp. 171, 175). These objects would be prioritized when people recognize a scene without strict time constraints [see Henderson and Hollingworth (1999) for a review]. Even though aerial images are very different from daily terrestrial images, this strategy of searching for diagnostic objects is likely to still be applicable. In fact, Loschky et al. (2015) suggested that aerial scenes may be categorized by image parts and individual objects. However, this strategy may not be equally useful for all aerial scenes. For example, airport scenes often contain airplanes that are diagnostic for that scene category while Industrial and School scenes share buildings and roads, making them difficult to distinguish from each other without more scrutiny. To study the impact of object-level information on influencing the eye movements for aerial scene categorization, we annotated critical objects in each image and used critical object saliency as object-level image statistic.

Furthermore, we investigated whether aerial viewing behavior systematically differs between different scene categories and which are the driving factors accountable for these differences. To this end, we conducted analyses at the scene category level. After determining the factors that contribute to aerial scene categorization, we were able to further study their tolerance for rotation. Based on the findings of Loschky et al. (2015, Experiment 2 & 3) that showed orientation-/rotational invariance in rapid aerial scene recognition, we expected to find similar tolerance for image rotation in the image statistics that influence this categorization process.

## Methods

### Participants

Twenty participants (eight females) with a mean age of 23.4 years (SD = 2.02) took part in this experiment. In studies of eye movements during scene perception, the numbers of participants vary from as small as 6 (Irwin & Zelinsky, 2002), 10 (Rayner et al., 2009) to 20 (Borji et al., 2013; Castelhano et al., 2009), or 24 (Oehlschlaeger & Võ, 2020) participants, or even more. In terms of an a priori power analysis, since there is a lack of previous results reporting the effect size of scene categories on aerial scene perception, we were not able to conduct an a priori power analysis (Oehlschlaeger & Võ, 2020). However, if we assume a medium effect size (Cohen’f = 0.25) and use a significance level of 0.05 and a statistical power of 0.8, the computed required sample size is 13. A sample size of 20 provides a statistical power of over 0.95. This calculation is based on G*Power (Faul et al., 2007). Hence, recruiting 20 participants would have successfully captured differences in eye movements for different aerial scene categories. All of them had normal or corrected-to-normal vision. Participants were naïve to the purpose of this study and were asked to observe images in order to be able to make scene categorizations. All of the participants were students or graduates of the major in remote sensing from the School of Remote Sensing and Information Engineering of Wuhan University. All would have had significant exposure to aerial viewpoint images during their studies. Participants provided informed consent after receiving complete instructions and explanations about the experimental procedure, with the knowledge that their anonymized data may be openly shared with others. This work was approved by the Natural Science Ethics Committee of Wuhan University.

### Stimuli and apparatus

**Fig. 1:**
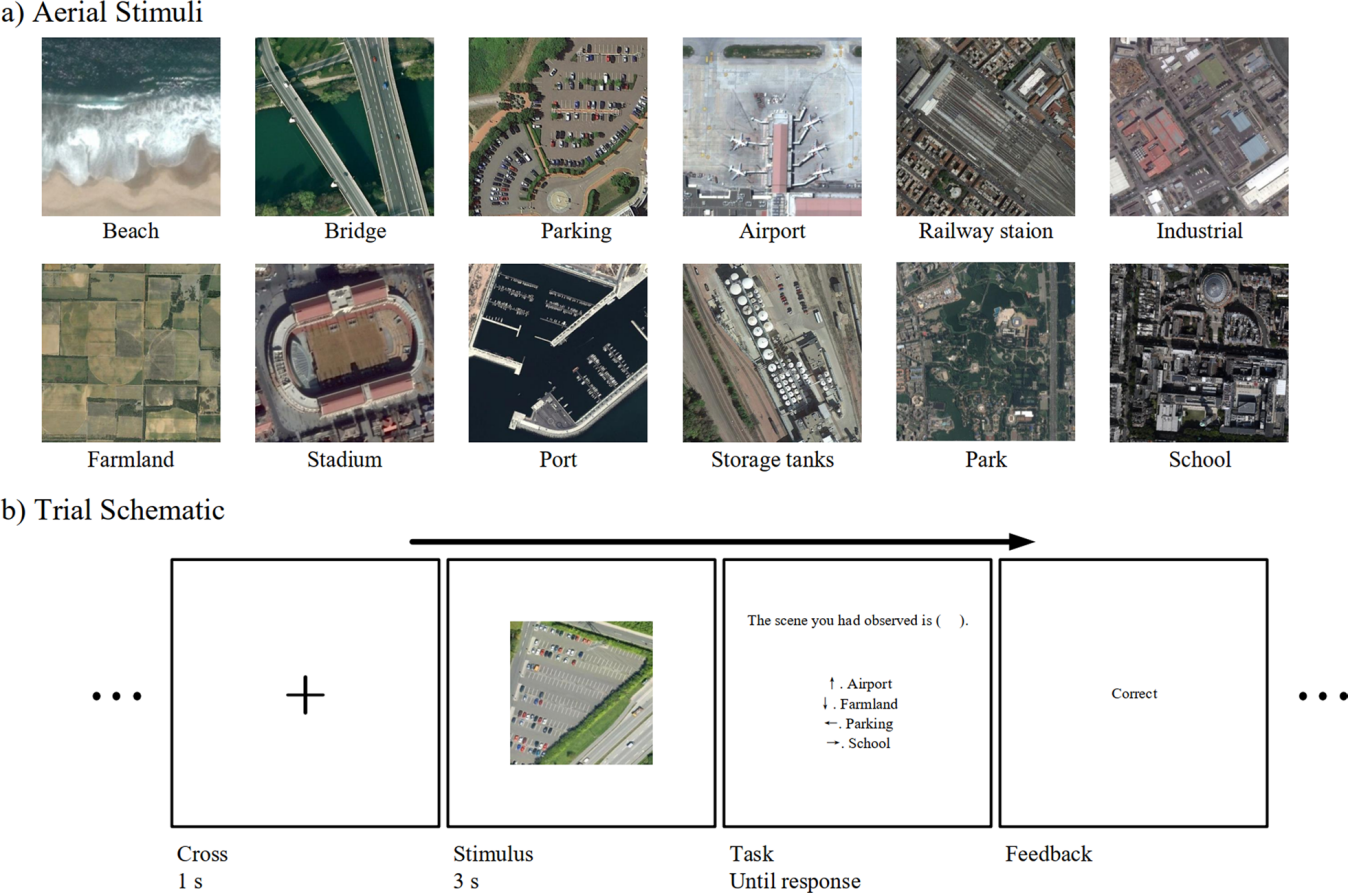
Stimuli exemplars and experiment setup. **A** A sample image is shown for each category. **B** Trial schematic. A fixation cross was shown for 1 s, followed by a stimulus presented for 3 s. A four alternative-forced choice task asked observers to categorize the observed scene. Feedback was given after response

Stimuli, as shown in Fig. 1a, were presented on a 19-inch Dell P1917S monitor, with a screen resolution of 1280 × 1024 pixels and a refresh rate of 60 Hz. Participants were instructed to observe the aerial stimuli and answer the question about scene categories, while sitting in front of the monitor at a distance of 60 cm, with their heads largely immobilized by the use of a chin rest for the duration of data collection. The seating distance resulted in about 40 pixels on the screen subtending one degree of visual angle (dva).

Eye movements of the left eye were recorded using the EyeLink 1000 Plus eye tracker (SR Research Ltd, Ontario, Canada), with a sample rate of 1000 Hz. The average accuracy of Eyelink 1000 Plus is reported to be 0.15° with 0.25–0.5° typical according to its user manual. Two nine-point target grid detection routines were used for calibration and validation of spatial accuracy of the eye tracking. Fixations and saccades were defined based on the saccade detection algorithm supplied by SR Research: saccades were identified by a minimum acceleration of 9500°/second and a minimum velocity of 35°/s.

For stimuli, we used a subset of the dataset Aerial Image Dataset (AID) (Xia et al., 2017). AID contains 10,000 true-colored aerial images in total for 30 categories and more than 200 images for each category. In this experiment, we chose 20 images from each of 12 categories, resulting in a total of 240 images. To mitigate the impact of potential individual differences in this task, categories were selected based on their relevance to everyday life (e.g., School and Parking) or suitability for effective learning with a limited set of exemplar images (e.g., Beach and Port). For each category, the first twenty images in the AID dataset were chosen. In occasional cases of image repetition or damage found during the stimuli screening progress, a randomly selected substitute image was used. All images were of 600 × 600 pixels, subtending approximately 15° in the horizontal and vertical directions.

### Procedure

The experimental design is shown in Fig. 1b. The experiment contained four blocks of sixty trials each. Before the formal experiment, participants had familiarized themselves with three images per category and achieved at least 90% accuracy on a 12-trial practice test. Stimuli used in practice were not presented in the formal test.

In the formal test, participants conducted eye-tracking calibration and validation processes prior to each block of trials. For a trial, a center cross appeared for 1 s followed by an aerial image presented at the center of the screen for 3 s. Upon stimuli offset, four category names, including the target category and three foils, were presented as possible responses. Participants were asked to choose the scene category of the aerial image using four arrow keys, one of which had been randomly mapped to the correct answer. The presentation sequence for each participant was randomly generated. There were five images per category per block presented in random order. Participants were instructed to maintain fixation on the cross before stimuli onset and to freely view presented images. A five-minute rest was enforced after each block.

### Data screening

Eye movements were recorded, starting 100 ms before the image onset. It is worth noting that trials on which first fixations had “drifted” were neither excluded nor treated differently as the literature suggests that central fixation bias in scene viewing is independent of the initial viewing point and serves as a viewing strategy (Tatler, 2007; Tseng et al., 2009). Since eye tracking started before an image onset, we deemed the recorded first fixation to be uninformative. Thus, with recorded first fixations excluded, all trials were further screened. Specifically, we considered a trial as valid if it met these criteria: (1) The participant categorized the scene correctly (causing exclusion of 2.12% of all trials). (2) The number of valid fixations was larger than two, meaning that, during the scene viewing, the participant shifted fixation rather than sticking to one single location (causing exclusion of 2.71% of all trials). (3) For each trial, if the eye tracker lost the track of eyes for more than 100 ms, the trial was considered invalid unless the gap could be attributed to a blink (removed just 1 trial). (4) We would have excluded any participant who lost eye tracking over 25% of the whole viewing time or where 25% of all trials was excluded (Cronin et al., 2020). No observers were excluded on this basis.

After screening the trials, we obtained 39,374 fixations across all participants. Next, fixations outside the image region were excluded (0.35% of all valid fixations). Fixations with a duration less than 100 ms (3.93%) or more than two standard deviations above the grand mean of all fixation duration (5.05%), which obeyed a log-normal distribution (M = 296 ms, SD = 139 ms), were also discarded. This data screening led to the discarding of 9.33% of all fixations from valid trials, leaving 35,699 valid fixations. Similarly, we pre-processed saccade data. Note that, unlike the first fixation, the first recorded saccade was taken into consideration. Any saccade onset within 75 ms of stimuli presented was removed as an error of anticipation (1.70% of saccades) (Cronin et al., 2020). If either the pre-saccadic or post-saccadic fixation was located outside of the image region the saccade was invalid (0.86% of saccades). The remaining 38,945 saccades were labeled as valid.

### Eye movement variables

Valid fixations of each image per participant were collapsed into a fixation location map (FLM). Center bias was quantified following Tseng et al.’s (2009) method, calculating the average distance of fixations to the image center. The values were normalized from zero (baseline condition with uniform fixation distribution) to 100 (all fixations precisely at the center). Standard error was estimated through 1000 bootstrap runs. A fixation density map (FDM) was calculated from convolving the FLM with a Gaussian kernel. The full width at half maximum of the Gaussian kernel was set at 0.5° (Le Meur et al., 2006), approximately 20 pixels, according to the reported accuracy in the EyeLink user manual.

To investigate the differences between viewing behavior for aerial images with different scene categories, we analyzed several eye-tracking parameters, including the mean fixation duration, the entropy of FDM, the number of fixations, the mean saccade amplitude, and gaze transition entropy (a measure of scanpath randomness, see below). Fixation duration was defined as the average duration of all fixations executed during image viewing. Entropy of the resulting FDM (called FDM entropy hereafter) for participant *p* observing image *I* was calculated using the standard MATLAB function (MathWorks, Inc.) according to Eq. (1), where *i* and* j* indicate pixels in the FDM of image *I* (Kaspar et al., 2013). The number of fixations was the fixation total during the 3-s viewing time. The mean saccade amplitude was defined as the average amplitude of all valid saccades during the image viewing. These variables were computed per observer per image. Then, the average of variable values across twenty observers represented the variable value for one image. For example, the number of fixations for one image was the mean of number of fixations across 20 observers. Similarly, these variable values for one scene category were the mean values across its twenty image samples. Thus, each scene category had 20 image samples, and each image has 20 observers.

1 $$\text{Entropy}_{I}^{p}=-\sum_{i=1}^{600}\sum_{j=1}^{600}{\text{FDM}}_{I}^{p}\left(i,j\right)\times log\left({{\text{FDM}}}_{I}^{p}\left(i,j\right)\right)$$

Gaze transition entropy (GTE) examines the overall uncertainty in determining the next fixation location provided the current fixation location (for a review, see Shiferaw et al., 2019). It considers spatial and temporal dependencies between two consecutive fixations and provides an overall estimation for the level of complexity or randomness in the pattern of visual scanning. Higher entropy suggests lower predictability. GTE was computed with Eq. (2). Given an image *I*, its spatial region is divided into *N* areas of interest (AOI). A vector **v** of length $N$ is produced where *v*_*i*_ is the probability of fixations falling into *N*^*th*^ AOI of image *I*. Fixation transition matrix **M** is produced where *M*_*i,j*_ is the probability of fixations transitioning from *i*^*th*^ to *j*^*th*^ AOI. Thus, **M** characterizes the rate of fixation transitions between AOIs. The observed GTE was then normalized by dividing it using the theoretical maximum entropy ${\text{GTE}}_{\max}={\log}_{2}\left(N\right)$. The benefit of using normalized GTE was discussed by Shiferaw et al. (2019). GTE was calculated for each image and each participant.

2 $${\text{GTE}}_{I}\left(M\right)=-\sum_{i=1}^{N}{v}_{i}\sum_{j=1}^{N}{M}_{i,j}\times {\log}_{2}\left({M}_{i,j}\right)$$

We split the image into $n=6$ equal segments in the horizontal and vertical directions, each subtending 2.5 dva, resulting in *N* = 36 AOIs. This parameter was used because the overall median saccade amplitude was 2.7 dva. Given that GTE considers two consecutive saccades, we believe that using the median avoids the bias toward longer or shorter saccades. Using different values of n (i.e., 5, 6, 8, or 10) had only a minor impact on the regression results. $n=6$ produced the best fitting results.

Overall, we consider five eye movement variables: *(1) fixation duration, (2) the number of fixations, (3) saccade amplitude, (4) the entropy of fixation density map, and (5) gaze transition entropy (scanpath randomness)*.

### Image features and statistics

At the low-level, we extracted energy-based features using Gabor filters with different orientations and spatial frequencies. They were used as a proxy for quantifying the early stage activation received by the visual system. Following the approach by Henderson et al. (2023), the Gabor filters comprised 12 unique orientations, linearly spaced between 0° and 360°. This collection of filters was applied in eight unique spatial frequencies that were logarithmically spaced between 0.35 and 8.56 cycles per dva. The notion of *population receptive field* (pRF) was introduced to account for the fact that a neuron receives a limited spatial range of stimulation (Dumoulin & Wandell, 2008). The pRF was described by a two-dimensional Gaussian response. Filter operation looped over a grid of candidate pRFs across images, producing a 96-dimensional activation vector at each pRF. One difference of our calculation from Henderson et al.’s (2023) was that we averaged the activations across pRFs in each dimension, focusing on the overall response rather than specific pRFs. This approach yielded a 96-dimensional activation vector for each image. Gabor features based on four lower spatial frequencies and four higher spatial frequencies were used separately as *Gabor responses on low S. F.* and *high S.F.* The low S.F. and high S.F. were averaged separately and were used as two low-level statistics.^1^

As shown by Loschky et al. (2015) and various practical applications of aerial image processing (He & Wang, 1990), texture is crucial in the identification of aerial scenes and objects. We applied Gray-Level Cooccurrence Matrix (GLCM) to analyze texture of aerial images (Lane et al., 2014). It calculates the number of pixel pairs with the same gray-level value for a given distance and direction (i.e., the offset) to reveal the texture and patterns present in the images. A concentration on diagonal line in GLCM means that the majority of pixels is of the same gray-level and thus the image is homogeneous. Consequently, it yields a greater value of homogeneity, which is a second-order statistic based on GLCM. We performed this in MATLAB (MathWorks, Inc.), with a grayscale quantification level of 64 and a 3-by-3 processing window (Lane et al., 2014). Overall, the mid-level feature and statistic for each image were its GLCM and homogeneity value, respectively.

Given that categorization process evolves to more complex stages (e.g., scene discrimination) at later period of visual processing (Harris et al., 2011; Rummukainen et al., 2014), we used the within- and across-category perceptual similarities (Within Sim. /WS and Across Sim. /AS) of images as two high-level image statistics. In this work, VGG-16 (Simonyan & Zisserman, 2014) was used to extract image features. As mentioned earlier, we conducted an eye movement experiment using a subset of 240 images from the AID dataset. These images were reserved as a test set for evaluation purposes. The remaining 3970 images from the twelve scene categories were divided into training and validation sets, containing 3176 and 794 images, respectively. Using the pretrained weights on the ImageNet dataset, we fine-tuned the network using the training data. This refinement yielded a satisfactory network for aerial scene classification with an accuracy of 93.75% in test. The output of block_5 from the network was extracted as the deep feature with 512 feature channels and a spatial resolution of $7 \times 7$ pixels. For each image, we calculated Within Sim. and Across Sim. statistics. WS was defined as the average Pearson correlation of deep features between the image and all other images from the same category. Similarly, AS was defined as the average Pearson correlation of deep features between the image and all other images from different categories. Note that only images that were correctly classified by the refined network were analyzed in this manner to minimize the impact of misclassified instances on the results.

Given that non-expert observers achieved a 68% true positive rate in searching for targets in aerial images (Rhodes et al., 2021) and diagnostic objects would be prioritized when people recognize a scene without strict time constraints (Henderson & Hollingworth, 1999), it seems likely that object-level information is readily accessible and plays a significant role in identifying aerial scenes during the relatively long viewing time used here (3-s). To test this hypothesis, we established criteria for defining critical objects at the scene category level. These criteria include: (1) The identity of a critical object can be unambiguously identified based on its low-level properties such as color, shape, orientation, size, etc. Thus, an airport terminal would be quite unambiguous, while a school building, while identifiable as a building might not be unambiguously a school; (2) the critical object or a combination of critical objects serves as a predictable identifier of a scene category. It is important to note that in our data not every category contains critical objects (e.g., School and Park), and a defined critical object for a scene category does not imply that this object must be present in every scene from that category (19 out of 20 Industrial images have blue roofs). We acknowledge that the definition of “critical objects” is somewhat subjective and differs in the aerial image and visual scene literatures. Thus, for example, a water area might be an “object” in an aerial image while being a texture or a substance in a terrestrial scene. Our list of critical objects for each scene category is available in Additional file 1: Table S1. For each image, two annotators with extensive experience of aerial image processing labeled a (visible and recognizable) target in the scene only if it was the defined critical object for that particular scene category. The experimenter explained the experiment to annotators and discussed with them the criteria for defining a critical object and the specific objects for every scene category. Agreements were reached. Critical objects were labeled using freeform polygons (86.11%) or rectangles (13.89%, for Farmland scenes and very small airplanes/tanks).

To quantify the influence of critical objects on the categorization process, we defined critical object saliency (COS) jointly considering the size and location of these objects. The size was defined as the ratio of the number of pixels enclosed by the polygon or rectangle annotating a critical object over the number of pixels of the image. Perceived size is a nonlinear, compressive function of physical area. According to Stevens’ power law, apparent size increases approximately with the 0.7 power of the area of the stimulus (Stevens, 1975, p. 54). This transformation was applied in this work. The location measured how close an object was to the image center. The distance from the mass point of a polygon/rectangle to image center was divided by the distance from image center to image corner, and then the ratio was subtracted from 1. The farthest four corners were indicated by a location value of 0 and the image center was 1. Then, the COS value for each image was calculated using the formula of Eq. (3), where $n$ denotes the number of critical object instances in the image. Only scenes with the whole image area as one critical object would produce a greatest saliency value of 1.

3 $${\text{Critical}} \;{\text{Object}}\; {\text{Saliency}}={\sum }_{c=1}^{n}{{\text{Size}}}_{c}\times {{\text{Location}}}_{c}$$

To expand the set of image statistics, we chose outputs from three specific VGG-16 layers (i.e., block_1, block_5, and fully connected layer 2) based on multicollinearity analysis (O’Brien, 2007; Ozturk, & Ullah, 2022). This selection was prompted by weak to strong correlations among image statistics in our data (refer to Additional file 1 for Multicollinearity Analysis details). We conducted principal component analysis (PCA) (Jolliffe & Cadima, 2016) on VGG-16 features, utilizing all resulting components with 100% variance explained. The L2-norms of PCA features were then computed as statistics for these layers, providing a meaningful distance metric in an orthogonal PCA space (Zalocusky et al., 2021). Analyses revealed that deeper VGG-16 layers extracted more scene category-relevant information. Thus, blk1, blk5, and fc2 were used as low-, mid-, and high-level image statistics, respectively, in this study.

Nine image statistics were considered in this work: *(1) Gabor response on low spatial frequencies, (2) Gabor response on high spatial frequencies, (3) homogeneity, (4) within-category perceptual similarity, (5) across-category perceptual similarity, (6) critical object saliency, (7–9) the L2-norms of PCA-transformed features based on outputs from VGG-16 (block_1, block_5, and fully connected layer 2)*. Low- and mid-level statistics were computed on grayscale images with dimensions of 240 × 240 pixels and 600 × 600 pixels, respectively. For the VGG-16 model, true color images with dimensions of 224 × 224 pixels were used. Image annotations were conducted on images with dimensions of 600 × 600 pixels, using CoLabeler.^2^ Since we defined no critical objects for Park and School scenes, their COS values were zeros. Scenes like Farmland and Beach contained critical objects covering a large proportion of the image area, resulting in greater saliency values.

### Implementations of regression and rotational invariance tests

Stepwise linear regression modeling was performed at both image and scene category levels using R Studio (version 2023.06.0 + 421, R version 4.3.1) (The R Foundation, Vienna, Austria). There were five dependent variables (i.e., eye movements) and each of them was regressed on nine predictors (i.e., image statistics). Model selection was based on Akaike information criterion (AIC) values and the stepwise regression used both backward and forward approach. Before the regression, luminance effect was controlled by regressing out the contribution of luminance from the eye movement data. Luminance was computed as the L component of the image in the CIE L*a*b* color space. The reason for this control is that image luminance values were not constant across the AID dataset (where our data was chosen from) and scene luminance influenced attention allocation during scene viewing as shown in other studies (e.g., Henderson et al., 2013). Subsequent steps of regression were based on the residual data after controlling luminance influence.

Original images (0°) were rotated counterclockwise at 90° intervals to produce images at 90°, 180°, and 270° orientations. Oblique angles were omitted due to computer analysis methods requiring input in the form of a horizontal rectangle. Otherwise, an oblique rectangle needs to be padded to create a minimum bounding horizontal rectangle. Such padding preserves the whole image space but introduces noise when calculating metrics that represent the whole image (e.g., homogeneity), or when pooling values from multiple pixels into a single one (e.g., in VGG-16).

The computation of image statistics for the rotated images followed the same process as that applied to the 0° images. Regarding the performance of the refined VGG-16 model classifying rotated images, the classification accuracies were 93.75%, 92.5%, 90.83%, and 93.75% at 0°, 90°, 180°, and 270°, respectively. While the model’s accuracy was lower than that of human observers (Mean: 97.88%) in this task, we argue that it was well trained and had learned useful rotation-invariant features for effective classification. Note that all 240 images were included in this analysis. As misclassified images differed among image orientations, restricting this analysis to only the correctly classified images for all orientations would lead to a reduction in the total number of images for certain categories (e.g., Industrial). This could potentially raise concerns related to statistical power. For each category, image statistics for different image orientations were subjected to One-way ANOVA tests, with image orientation as the factor.

## Results

### General eye movement patterns

For a summary of specific values of the five eye movement variables and the nine image statistics for each scene category, along with the One-way ANOVA results with scene category as a main effect, please see Additional file 1: Table S2 and S3.

**Fig. 2:**
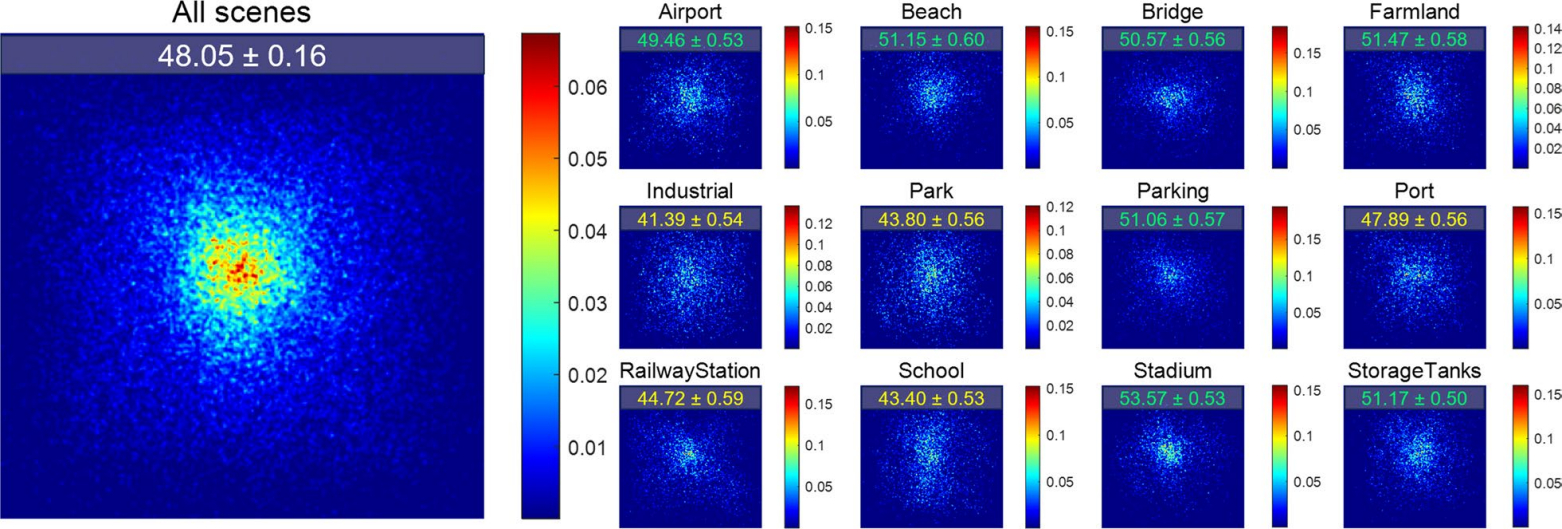
Illustrations of fixation locations in a dimension of 600 × 600 pixels. The left larger plot contains fixations from all stimuli. The right twelve smaller plots are the results for each scene category. Colorbars indicate the density of each plot. Each plot has its own scale because they were normalized independently to sum to one over pixels. On the top of each plot shows the measured center bias (mean ± SD) where a maximum value of 100 means that all fixations are on the center of images and a minimum value of 0 means fixations are of a uniform distribution. Some categories induced more (green) or less (yellow) centered viewing bias than measured using all scenes (white)

Initially, we visualized the fixation points for all stimuli as well as for the stimuli of each category separately. The data for each category comprised fixations across all participants from all images belonging to that category. The fixation point maps were convolved using a Gaussian kernel with a full width at half maximum of 1 pixel, for a clearer pattern demonstration. Figure 2 shows an overall center bias when observers viewed aerial images. Two reasons can be offered for the observed center bias. First, human observers are inclined to start from and be more attentive to the center of an image when observing it (Tatler, 2007; Tseng et al., 2009). Second, the scene categorization task might induce the image collectors to arrange scene-relevant information close to the center of an image, a phenomenon sometimes called “photographer bias” as a source of viewing center bias (Tseng et al., 2009). In Fig. 1, for example, look how the terminal is located at the center of the image even though it could be located anywhere. In these senses, the observed viewing center bias should not be simply attributed to the pre-stimuli center cross.

**Fig. 3:**
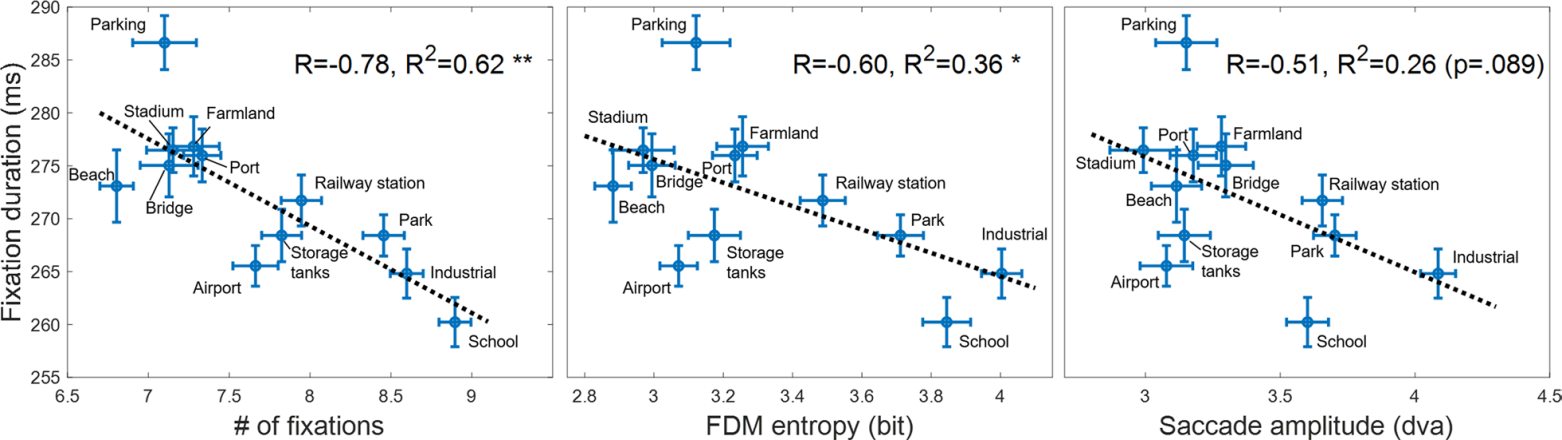
Fixation duration as a function of the number of fixations (left), FDM entropy (middle), and saccade amplitude (right), respectively. In each plot, a dot is for a scene category and error bars show the $\pm 1$ SEM across image samples from individual category. Dotted lines are fitting results of linear regression, along with Pearson correlation coefficients (R), R squares and statistical significances (**p < 0.01, *p < 0.05)

Notably, fixation location patterns varied across scene categories. First, center bias was quantified (see Methods). As shown by the numeric text labels on the individual panels of Fig. 2, the degree of viewing center bias was more profound for some categories (green) while weaker for others (yellow). Other eye movement measurements also differed across categories. As shown in Fig. 3, a dot indicates a scene category and error bars shows $\pm 1$ Standard Error of Mean across image samples from each category. Lines were fitting results of linear regression, along with R squares and statistical significances. Within the 3-s time limit, observers dynamically adjusted their strategy of identifying aerial scenes, balancing between more areas explored and more details collected. Specifically, a greater number of fixations, higher FDM entropy values, and larger saccade amplitudes were linked to shorter fixation durations. Statistical tests showed that the number of fixations was most predictive of fixation duration (R^2^ = 0.62, *p* = 0.002), followed by FDM entropy measure (R^2^ = 0.36, *p* = 0.038). The predictive power of saccade amplitude to fixation duration achieved a marginal significance (R^2^ = 0.26, *p* = 0.089). This marginal effect is increasingly viewed as evidence for hypotheses (Pritschet et al., 2016).

These negative correlations between fixation duration and each of the other three measurements seem to result from the viewing time limit. That said, using more fixations naturally compresses individual fixation durations. It might be interesting to think about these results in terms of explore/exploit trade-off language. The distinction between exploration and exploitation has been used in domains from decision-making studies in animal foraging (Mehlhorn et al., 2015) to human betting behavior (Navarro et al., 2016). In our work, participants could be actively foraging for targets such as critical objects.

Among these five eye movement measures, gaze transition entropy (GTE) was not correlated with any of the other four (*ps* > 0.241). As indicated by the GTE values (0.072–0.088; Additional file 1: Table S2), the unpredictability of participants’ fixation sequences accounts for about 8% of the theoretical maximum unpredictability, which means that the current fixation has a relatively deterministic relationship to the next fixation. This might suggest that eye movements for categorizing aerial images are generally organized and predictable. Alternatively, it might just be a side effect of the strong center bias observed in our data and the $6\times 6$ partition of image space in GTE calculation.

### Image statistics predict eye movements

Above results showed that participants’ viewing behavior during aerial scene categorization varied significantly between different scene categories. To study the influential factors underlying this cognitive process, we then investigated which features contributed to the identification of aerial scenes at both the image and scene category levels. According to our hypothesis, the availability of critical objects in aerial scenes impacts eye movements during this process. In addition, other factors are likely to simultaneously influence observers’ viewing, such as texture and perceptual similarity.

First, we performed stepwise linear regression modeling at the image level using residual data after controlling the potential influence of image luminance (see Methods). Results are summarized in Table 1. All regression models were statistically significant (*ps* < 0.001). Mid- and object-level image statistics were largely predictive of the eye movement patterns. PCA-transformed VGG-16 image statistics were strongly associated with various eye movement variables in this study. Since a support vector machine can be well trained with block_5 (91.54%) and FC_2 features (94.45%) to classify aerial scenes, we argue that scene category-relevant knowledge is represented in those layers. Therefore, it might suggest that in aerial scene categorization, scene category-level information itself plays a key role. Excluding COS values of 0 yielded similar regression outcomes.

**Table 1:** Summary of regression models at the image level

Estimate *t* value	Std. Error *p* value	Coefficient information (Organized as shown to the left; Intercept omitted)									
Image statistic		Eye movement									
		Fixation duration		Number of fixations		FDM entropy		Saccade amplitude		GTE (Scanpath randomness)	
Low	Low S.F			10.20 2.02	5.06 *					0.18 2.36	0.08 *
	High S.F					10.56 2.20	4.80 *				
	Block_1 PCA	6.4e−03 2.45	2.6e−03 *	− 3.3e−04 − 2.01	1.6e−04 *	− 3.3e−04 − 3.09	1.1e−04 **	− 1.9e−04 − 2.04	9.5e−05 *		
Mid	Homogeneity			− 1.99 − 3.20	0.62 **	− 1.46 − 3.41	0.43 ***	− 1.56 − 4.52	0.35 ***	4.4e−02 4.66	9.5e−03 ***
	Block_5 PCA			− 8.3e−04 − 2.62	3.2e−04 **	− 7.2e−04 − 3.91	1.8e−04 ***	− 1.0e−03 − 4.46	2.3e−04 ***	1.5e−05 2.89	5.3e−06 **
High	Within Sim					− 0.73 − 1.80	0.41 = 0.073	− 1.61 − 3.22	0.50 **		
	Across Sim					2.06 1.98	1.04 *				
	FC_2 PCA			4.9e−03 2.05	2.4e−03 *	5.1e−03 3.4	1.5e−03 ***	6.0e−03 3.37	1.8e−03 ***	− 1.3e−04 − 3.36	4.0e−05 ***
Object	Critical object saliency	12.85 5.05	2.54 ***	− 0.96 − 5.47	0.18 ***					− 1.2e−02 − 4.42	2.7e−03 ***
Adjusted R^2^		0.15 (***)		0.42 (***)		0.35 (***)		0.20 (***)		0.16 (***)	

***p < 0.001, **p < 0.01, *p < 0.05

**Fig. 4:**
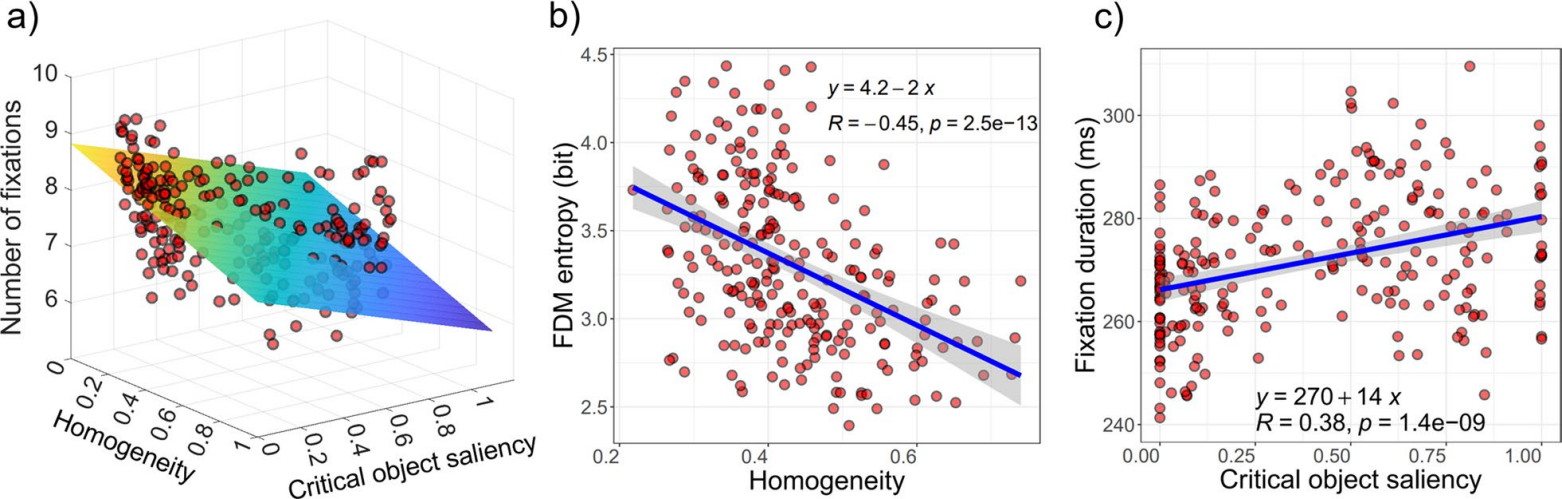
Illustrations of the relationships between eye movement variables and image statistics. In all plots, each red dot represents one image. In (**a**), the number of fixations as a function of homogeneity and COS is shown, as well as the linear fitting plane. In (**b**, **c**), FDM entropy and fixation duration are shown as functions of homogeneity and COS, respectively. R indicates the Pearson correlation coefficients of two variables in each plot

Three plots in Fig. 4 show the relationship between image statistics and eye movements. For illustration purpose, only a subset of relationship between variables is demonstrated. These scatterplots show the direct relationship between the mentioned pairs of variables without accounting for any additional factors or predictors that might influence the relationship such as those in the regression models. Specifically, more homogeneous scenes led to fewer fixations and lower FDM entropy, indicating that a smaller image area was explored (Fig. 4a & 4b). Lower COS in aerial scenes induced more fixations and shortened fixation durations during viewing (Fig. 4a & 4c).

Regression results at the scene category level are shown in Table 2. It is worth noting that using nine independent variables to regress one response variable of 12 samples (i.e., 12 scene categories) suffers from power issues. To partly address this, we reported Adjusted R^2^, as it penalizes the inclusion of variables that do not improve the model and takes into account the number of predictors and the sample size in the model. Additionally, the scene category-level analyses were reported despite the potential power issues for two reasons: 1) Statistical tests indicate significance; 2) consistent relationships between eye movements and image statistics exist at both image and scene category levels, as indicated by the same signs ( ±) of estimate values (except for the one with Gabor Low S.F.), suggesting a stable impact of these features on aerial viewing behavior. Thus, we argue that at the scene category level, image statistics that impacted aerial viewing behavior mainly came from higher- and object-level information.

**Table 2:** Summary of regression models at the scene category level

Estimate *t* value	Std. Error *p* value	Coefficient information (Organized as shown to the left; Intercept omitted)									
Image statistic		Eye movement									
		Fixation duration		Number of fixations		FDM entropy		Saccade amplitude		GTE (Scanpath randomness)	
Low	Low S.F			*− 19.49* *− 2.23*	*8.7* *= 0.076*						
	High S.F									*− 1.12*﻿ *− 2.17*	*0.51* *= 0.073*
	Block_1 PCA										
Mid	Homogeneity							− 1.74 − 2.92	0.60 *		
	Block_5 PCA			− 1.8e−03 − 4.5	3.9e−04 **	− 1.7e−03 − 4.22	3.9e−04 **	− 0.002 − 4.86	0.0004 **	*1.8e−05* *2.41*	*7.4e−06* *= 0.053*
High	Within Sim							− 2.24 − 2.73	0.82 *		
	Across Sim			7.03 2.81	2.51 *						
	FC_2 PCA			2.5e−02 4.93	5.0e−03 **	0.014 3.26	0.004 *	0.017 3.872	0.004 **		
Object	Critical object saliency	14.31 3.72	3.85 **	− 1.32 − 6.92	0.19 ***	− 0.47 − 2.87	0.16 *			− 0.018 − 3.09	6.0e−03 *
Adjusted R^2^		0.66 (*)		0.96 (***)		0.82 (**)		0.81 ﻿(**)		*0.57* (*p* *= 0.062)*	

***p < 0.001, **p < 0.01, *p < 0.05

**Fig. 5:**
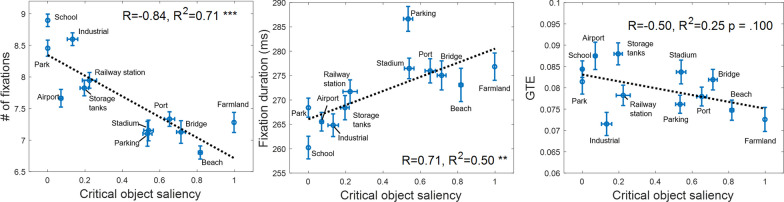
From left to right, the number of fixations, fixation duration, and GTE (scanpath randomness) are plotted as functions of critical object saliency. Each dot is for one category. Error bars indicate the $\pm 1$ SEM across image samples within that category. Lines are fitting results of linear regression, along with Pearson correlation coefficients (R), R squares, and statistical significances (**p < 0.01, *p < 0.05). On a scene category basis, FDM entropy was correlated with COS significantly (p = 0.025), saccade amplitude (*p* = 0.091), and GTE (*p* = 0.100) were marginally correlated with COS

Figure 5 shows the number of fixations (5a), fixation duration (5b), and GTE (5c) as functions of critical object saliency. The category-level viewing patterns partly resembled image-level results. When an image was from a scene category containing fewer or no salient critical objects (e.g., School and Industrial), observers’ viewing behavior was systematically more exploratory, and slightly more random. The results from both the category and image levels supported our hypothesis that the availability of critical objects influences eye movements when categorizing aerial scenes.

### Multicollinearity and non-exhaustive image statistics

Some image statistics we used were correlated with each other (Additional file 1: Fig. S2). There is an asymmetry in this multicollinearity issue (Belsley et al., 2005; O’Brien, 2007): if the ${i}^{th}$ regressor is statistically significant even if it has considerable multicollinearity, it is statistically significant in the face of that collinearity. However, if the *i*th regressor has a large amount of multicollinearity and turns out to be a nonsignificant predictor, this may be the situation where collinearity has a negative effect. Thus, we think the correlations among these selected image statistics would not contradict their predictive power as detected, especially after we had controlled the multicollinearity. Given that *Gabor High S. F.* showed severe multicollinearity (Multicollinearity analysis in the Additional file 1), one might attribute its lack of detected predictive power in regressions to this. It is possible. However, *Gabor Low S. F.*, while less collinear than the other three VGG-16 L2-norm based image statistics, still poorly predicted eye movements. Thus, Gabor responses may be truly insufficient to capture aerial viewing patterns.

While other image statistics might also predict aerial eye movement patterns, the relatively broad predictor range in this study yielded reasonable and interpretable outcomes. We argue that the results are not mere artifacts of non-exhaustive predictors. Further research may uncover additional image statistics contributing to aerial visual attention in this context.

### Rotational invariance of image statistics

Rapid aerial scene categorization has been found to depend on rotation-invariant information (Loschky et al., 2015). Obviously, image rotation has no impact on the size of an object in an image or its distance from the image center. Thus, we tested whether other image statistics we analyzed in this work are sensitive to rotational variations. Results showed that nine image statistics at four image orientations (0°, 90°, 180°, and 270°) are not statistically different (One-way ANOVA tests, *ps* > 0.187) or marginally different (Within Sim. of Beach, *p* = 0.064). Why Within Sim. of Beach scenes were more affected by image rotation is not clear. It might be because the used Beach stimuli had their water bodies more frequently appear in the lower half of the image, which could have biased model training. When images were rotated, water bodies were relocated to areas originally occupied by sandy lands, possibly introducing noise in feature extraction. Note that the rotational invariance test for Gabor features is trivial in this work, as Gabor responses were averaged across twelve Gabor filter orientations (i.e., 0° to 165° at a step of 15°).

## Conclusions from results

In this work, the relationships between viewing behavior for aerial scene categorization and image statistics was examined. Twenty experienced subjects’ eye movements were recorded while they categorized aerial scenes. A general center bias in viewing was observed. Eye movement patterns varied among scene categories in terms of fixation durations, the number of fixations, the entropy of fixation density map, saccade amplitudes, and gaze transition entropy (scanpath randomness).

Results showed that viewing behavior was more exploratory when (1) an image featured a less homogeneous texture, and/or (2) when the image contained few or no salient objects that could provide category-diagnostic information. VGG-16 based image statistics were strongly correlated with viewing patterns, suggesting that other image features from low- to high-level impacted visual attention in this task. However, the exact features, corresponding to those VGG-16 layers, are not entirely known. At the scene category level, higher-level image statistics and critical object saliency were found to be more predictive of viewing behavior. Scanpaths were generally organized, showing minor differences across categories. These differences could still be roughly captured by critical object saliency. Participants were inclined to fixate on defined critical objects (Additional file 1: Fig. S3). The image statistics tested in this study were rotation-invariant. In summary, our results supported our hypothesis that the availability of critical objects influences scene sampling and overt visual attention in aerial scene categorization.

## General discussion

### Why objects are emphasized in aerial scenes

The impact of critical objects on eye movements during aerial scene categorization may be also attributed to the fact that most scene categories we used were man-made. Man-made scenes are defined by their functions in contrast to natural scenes that are defined more by their appearance/texture. Lloyd et al. (2002) referred to these man-made scenes as lower-order, or more specific categories. Two man-made scenes can look similar while serving different purposes. For instance, distinguishing between commercial and industrial land uses can be difficult if relevant contextual scene information is not available (e.g., in an aerial image the shop signs are seldom visible and building height information is limited). Searching for critical objects representing functional purposes may be the most efficient way to determine land use for man-made scenes, and, perhaps, more generally. When such functionally meaningful objects are absent, scenes that share similar-looking objects but belong to different scene categories can produce categorization errors. This is evidenced by Loschky et al.’s work (2015), showing that compared to natural aerial scenes, subjects made more categorization confusion between man-made aerial scenes no matter the scenes were either intact or texturized images.

Our experimental use of a 3-s viewing time may have contributed to the observed attentional emphasis on local regions and objects. Without strict time constraints, diagnostic objects were prioritized when people had to recognize a scene (Henderson & Hollingworth, 1999). It would have been possible to use briefer presentation times, as prior research has shown that observers can identify aerial scene categories with just ~ 300 ms or shorter presentation. Under those short-viewing conditions, a global-to-local bias is observed and categorization becomes dependent on low-frequency information rather than on selective attention to any diagnostic objects (Schyns & Oliva, 1994). One might expect similar results with briefly presented aerial images though, of course, eye movements become less useful and other methods would be needed to analyze the data.

Global information like spatial relationships that are available from low-pass filtered images is believed to be useful enough in rapid scene analysis (Oliva & Torralba, 2001, 2006; Sanocki, 2003; Schyns & Oliva, 1994; Wilder et al., 2018). Others argued that localized information is at least as essential as global information in scene categorization (Loschky & Larson, 2008; Vogel et al., 2007). Recently, Wiesmann and Võ (2022) have demonstrated that global scene properties are useful for scene categorization with above-chance level performance, but fast, effortless, and high-accuracy performance requires local, high-resolution information such as objects. Indeed, observers can efficiently report object-level information in a single glance of grayscale images (Fei-Fei et al., 2007). In our work, we verified the importance of critical objects in influencing viewing behavior for aerial categorization. In addition, participants preferentially fixated on critical objects (Additional file 1: Fig. S3). They also showed longer RTs for scenes of lower COS values (Additional file 1: Fig. S4) and reported greater categorization difficulties for Park and School scenes (Additional file 1: Fig. S5). These results align with Loschky et al.’s (2015), suggesting that aerial scenes may be categorized based on their parts and individual objects.

The combination of the effects of critical objects and image homogeneity on aerial viewing behavior may indicate that aerial scene categorization is based on both specific object recognition and broader scene perception mechanisms. This would be consistent with Groen et al.’s (2017) argument that low- and mid-level properties may be particularly diagnostic for scene perception and high-level properties for object recognition. To be clear, while we stress the key role of critical objects in aerial scene viewing behavior and in aerial scene analysis, we are not arguing that specific objects are indispensable for categorizing aerial scenes. Observers can effectively categorize aerial images even in the absence of critical objects (Additional file 1: Fig. S1), but, all else being equal, observers seem to make more use of specific object information in our aerial scene task than they might in a terrestrial task.

It might be interesting to examine this issue with briefly presented stimuli. The ability to detect object-level information from a brief glance of daily images has not been extensively studied with aerial images. This raises a possibility that humans emphasize objects but are less efficient at detecting and recognizing them in aerial scenes, so they perform more poorly (e.g., Loschky et al., 2015) and might need longer processing times compared to terrestrial scenes.

### Categorical guidance and implications for automation

During image perception, category-relevant information is building up even if observers are merely told to memorize the images (Damiano et al., 2019; Long et al., 2018). Interestingly, both of these studies found the effects of mid-level visual features, either on the level of organized response elicited from object-selective cortex along the entire ventral pathway (Long et al., 2018) or on the guidance of fixations in a more top-down, categorical-specific way during the viewing (Damiano et al., 2019). The present work also supports the effectiveness of mid-level features in scene processing by showing that homogeneity significantly influenced image-level viewing patterns in this task.

The development of Artificial Intelligence has helped people in various trades reduce the burden of processing massive volumes of aerial images. Most existing methods of automated aerial image processing (e.g., Dimitrovski et al., 2023; Kotaridis & Lazaridou, 2021) exploited low- to high-level deep features without specifying categorical information during feature extraction. However, four facts deserve more attention in thinking about future development of AI systems: 1) Because humans can rapidly categorize scenes and/or extract scene gist (Loschky et al., 2015; Oliva, 2005), it is likely that the later period of viewing can be strongly biased by a preliminary assessment of the scene that is quite likely to be correct. 2) Even in category-irrelevant tasks, category-selective information modulates neuronal responses in the human brain (Long et al., 2018) and guides human visual attention (Damiano et al., 2019). 3) Experts make sophisticated use of semantic information to perform memory tasks using aerial images (Lansdale et al., 2010; Šikl et al., 2019). Finally, 4) as we revealed in this work, observers’ visual attention patterns vary across different aerial scene categories. Future development of automated systems could benefit from the adoption of these aspects of human processing of aerial images. These advances could, in turn, prove beneficial in designing and evaluating automated decision-support systems (e.g., Deepak & Ameer, 2021; Barata et al., 2023) and facilitate cognitive research (e.g., Agudo et al., 2024; Liu et al., 2020; Xu & Vaziri-Pashkam, 2021).

### Invariance in aerial viewpoints

Our rotational invariance analyses echo Loschky et al.’s (2015), finding that the information aiding rapid aerial scene categorization was rotation-invariant. With the task of aerial image memorization, however, image rotation led to a decline in accuracy for both experts and non-experts (Šikl et al., 2019). This contrast between the two tasks’ results suggests that, while categorization benefits from information that remains consistent across aerial viewpoints, memory recall is susceptible to the alterations introduced by image rotation. Whether image rotation has an impact on eye movement patterns for aerial tasks remains unknown. When a task requires processing viewpoint-sensitive information, there might be different eye movement patterns across image orientations. Further investigation into these potential rotation-induced variations in eye movements in various aerial tasks could probe deeper into the complex interrelation between perception, cognition, and the interpretation of aerial images.

The relative rotational invariance of aerial scene categorization may be a disadvantage when it comes to visual search (see Sanocki, 2003). In terrestrial imagery, a target like a building might be more likely to appear in the lower half of the image (depending on viewpoints). This can aid search. In aerial images, such regularities do not occur. Some spatial relationships will remain useful in overhead imagery. For example, search is can be guided by “anchor objects”. The toothbrush is likely to be near its anchor, the sink (Boettcher et al., 2018). An aerial context, search cars, is more profitably anchored to roads than to rivers. However, the direction from anchor to target object is likely less constrained in an aerial image than in a terrestrial one, where, for instance, the computer monitor is likely to be not just near, but reliably above the surface of the desk.

### Limitations

This study has certain limitations. Regression analyses at the scene category level likely suffered from a lack of statistical power. Unlike terrestrial images, aerial images typically offer a broader field of view in a single frame. In this work, we presented aerial scene stimuli in isolation, following the approach of previous studies. To explore how different scenes or categories interact and affect the perception of these images, it might be beneficial to use stimuli that feature multiple scene categories or multiple instances of one category in the frame of one image. In addition, it is possible that our eye movement statistics are “contaminated” by task-irrelevant eye movements. We fixed the viewing time as 3-s for each image. If observers conclusively categorized a scene after a fraction of a second, they might have been doing some completely different task (or no task at all) for the remaining time. In the future work, it might be useful to allow observers to freely control the pace of trials, ending a trial as soon as the response was generated. The resulting response time measure could also shed new insight on human viewing of aerial images. For example, Lloyd et al. (2002) found that larger aerial photographs were associated with more accurate and confident categorization but not faster responses. Moreover, a self-paced viewing paradigm would better mimic real-life viewing conditions and behaviors.

### Supplementary Information

**Additional file 1.** Supplementary Tables and Figures.

## Data Availability

Data, analysis scripts, and stimuli are available at https://osf.io/4n3rc/.

## Footnotes

1 The low/high spatial frequency partition produced the best regression results compared to the vertical/horizontal/oblique partition, or the grant mean of the 96-dimensional vector.

2 An open-source annotation tool, available at http://www.colabeler.com/.
