# Supplementary material for: Toward viewing behavior for aerial scene categorization
Source: Cogn Res Princ Implic. 2024 Mar 26;9:17. doi: 10.1186/s41235-024-00541-1 (PMC10965882; doi:10.1186/s41235-024-00541-1)
Supplement: Supplementary file 1 — Additional file 1. Supplementary Tables and Figures. [file 41235_2024_541_MOESM1_ESM.docx]

# **Supplemental Materials**

**Table S1** The list of defined critical objects for each aerial scene category

| Aerial scene category | Critical object(s) |
| --- | --- |
| Airport | Airplane |
| Beach | Water body, sandy land |
| Bridge | Water body, road |
| Farmland | Green and soil-colored surface |
| Industrial | Blue roof (Sun et al., 2020) |
| Park | NONE (Probably need inferences about objects) |
| Parking | Car, parking slot |
| Port | Ship, water body |
| Railway station | Railroad |
| Stadium | Sport venue |
| School | NONE (Probably need inferences about objects) |
| Storage tanks | Tank |

**Table S2** Eye movement values for each scene category. Mean (± SEM). Maximum values are marked in red and minimum values in blue. The bottom row shows *F*-statistics and significance level of One-way ANOVA with scene categories as main effect (*** *p* < .001).

| **Category** | **Fixation duration (ms)** | **Number of fixations** | **FDM entropy (bit)** | **Saccade amplitude (dva)** | **Gaze**  **Transition Entropy (bit)** |
| --- | --- | --- | --- | --- | --- |
| Airport | 265.54 (±1.92) | 7.662 (±.140) | 3.071 (±.054) | 3.078 (±.098) | .087 (±.003) |
| Beach | 273.09 (±3.42) | **6.804 (±.103)** | **2.882 (±.053)** | 3.115 (±.093) | .075 (±.002) |
| Bridge | 275.04 (±2.98) | 7.128 (±.179) | 2.994 (±.067) | 3.298 (±.102) | .082 (±.002) |
| Farmland | 276.84 (±2.80) | 7.280 (±.159) | 3.256 (±.074) | 3.282 (±.090) | .073 (±.003) |
| Industrial | 264.82 (±2.32) | 8.597 (±.102) | **4.003 (±.058)** | **4.086 (±.065)** | **.072 (±.003)** |
| Park | 268.42 (±1.95) | 8.455 (±.128) | 3.711 (±.066) | 3.702 (±.079) | .081 (±.003) |
| Parking | **286.64 (±2.55)** | 7.100 (±.197) | 3.122 (±.098) | 3.151 (±.114) | .076 (±.002) |
| Port | 275.98 (±2.49) | 7.333 (±.115) | 3.233 (±.064) | 3.177 (±.086) | .078 (±.002) |
| Railway station | 271.72 (±2.41) | 7.946 (±.125) | 3.487 (±.065) | 3.656 (±.075) | .078 (±.002) |
| School | **260.23 (±2.33)** | **8.896 (±.099)** | 3.845 (±.069) | 3.601 (±.078) | .084 (±.002) |
| Stadium | 276.48 (±2.11) | 7.153 (±.164) | 2.969 (±.089) | **2.992 (±.124)** | .084 (±.003) |
| Storage tanks | 268.42 (±2.48) | 7.824 (±.123) | 3.174 (±.075) | 3.144 (±.097) | **.088 (±.003)** |
| ANOVA (F, p) | 7.79 (***) | 23.27 (***) | 26.91 (***) | 12.58 (***) | 4.63 (***) |

**Table S3** Image statistic values for each scene category. Mean (± SEM). Maximum values are marked in red and minimum values in blue. The bottom row shows *F*-statistics and significance level of One-way ANOVA with scene categories as main effect (*** *p* < .001).

| **Category** | **Gabor:**  **low spatial frequency** | **Gabor:**  **high spatial frequency** | **Homogeneity** | **Within Similarity** | **Across Similarity** | **Critical Object Saliency** | **Block_1 PCA** | **Block_5 PCA** | **FC_2 PCA** |
| --- | --- | --- | --- | --- | --- | --- | --- | --- | --- |
| Airport | .086 (±.002) | .065 (±.001) | .422 (±.010) | .227 (±.005) | .128 (±.003) | **.071 (±.009)** | 1378.6 (±48.9) | 1137.0 (±31.4) | **104.8 (±2.2)** |
| Beach | .071 (±.003) | **.046 (±.001)** | **.584 (±.022)** | .367 (±.006) | **.056 (±.003)** | .817 (±.010) | 998.5 (±19.2) | 1034.6 (±38.8) | 115.8 (±6.8) |
| Bridge | .082 (±.002) | .053 (±.001) | .545 (±.014) | .279 (±.006) | .100 (±.004) | .714 (±.024) | 1317.2 (±42.8) | 1281.6 (±32.9) | 125.4 (±4.8) |
| Farmland | **.069 (±.002)** | .050 (±.001) | .479 (±.013) | **.386 (±.011)** | .123 (±.004) | **.998 (±.001)** | **978.3 (±14.4)** | 1076.4 (±20.9) | 105.8 (±2.9) |
| Industrial | .094 (±.002) | .070 (±.001) | .388 (±.007) | .290 (±.009) | .128 (±.003) | .133 (±.028) | 1248.8 (±42.0) | 1089.1 (±34.3) | 134.4 (±5.5) |
| Park | .085 (±.002) | .065 (±.002) | .391 (±.011) | .311 (±.009) | .120 (±.004) | 0 (NAN) | 1213.8 (±399.7) | 1254.1 (±49.0) | 124.0 (±5.3) |
| Parking | .1035 (±.003) | .081 (±.002) | .367 (±.016) | .307 (±.013) | .089 (±.007) | .534 (±.025) | **2078.9 (±166.7)** | 1437.9 (±62.8) | 139.0 (±9.3) |
| Port | .104 (±.003) | .072 (±.003) | .481 (±.029) | .265 (±.009) | .113 (±.004) | .652 (±.027) | 1938.8 (±107) | 1547.1 (±50.8) | 153.9 (±8.5) |
| Railway station | .089 (±.002) | .069 (±.002) | .348 (±.014) | .250 (±.007) | **.130 (±.004)** | .224 (±.038) | 1388.0 (±73.3) | **1029.3 (±25.7)** | 108.5 (±2.9) |
| School | **.1040 (±.002)** | **.083 (±.002)** | **.320 (±.011)** | .380 (±.012) | .126 (±.002) | 0 (NAN) | 1780.2 (±58.4) | 1326.7 (±31.9) | 154.4 (±5.6) |
| Stadium | .097 (±.003) | .058 (±.002) | .458 (±.015) | .341 (±.015) | .114 (±.004) | .540 (±.026) | 1356.1 (±70.8) | 1392.3 (±45.0) | 132.4 (±5.1) |
| Storage tanks | .103 (±.002) | .075 (±.002) | .373 (±.010) | **.224 (±.006)** | .109 (±.005) | .196 (±.023) | 1587.7 (±60.3) | **1668.1 (±56.2)** | **160.8 (±10.3)** |
| ANOVA (F, p) | 27.26 (***) | 42.20 (***) | 28.01 (***) | 36.80 (***) | 30.17 (***) | 257.37 (***) | 22.20 (***) | 25.14 (***) | 9.66 (***) |

## **Categorization accuracy**

**Fig. S1** Confusion matrix of aerial scene categorization. Performance for each category was satisfactorily good. The accuracy drop for Park and Parking was probably caused by the visual confusion between the spellings of “Park” and “Parking” for native Chinese speakers. In fact, one participant mentioned that sometimes they mistakenly took Park as Parking, or otherwise. This is evidenced by the confusion matrix. However, since the number of such trials were small and the specific affected trials could not be determined, we did not treat them differently.

## **Multicollinearity analysis**

Variance Inflation Factor (VIF) measures how much variances of the estimated regression coefficients are increased over the case of no correlation among *p* regressors. It is calculated as $VIF=\frac{1}{1-R_{i}^{2}}$, where $R_{i}^{2}$ is the squared multiple correlation of the $i^{th}$ independent variable regressed on the other independent variables in the analysis. If ${VIF}_{i}$ is larger than a threshold value of 10, it is deemed to have severe or serious multicollinearity and thus removed from regression (O’Brien, 2007; Ozturk, & Ullah, 2022). Calculation is based on the ‘vif’ function from the R 'car' package (Fox & Weisberg, 2019). *Gabor High S. F.* is reserved as it is the counterpart of *Gabor Low S.* “Qualified” predictors are (in parentheses are VIFs):

1. Blk1 PCA (5.32),
2. Blk5 PCA (4.87),
3. FC2 PCA (4.57),
4. Gabor Low S. F. (4.65),
5. Gabor High S. F. (13.53),
6. homogeneity (3.88),
7. within Sim. (1.50),
8. across Sim. (1.44),
9. Critical Object Saliency (2.42)

**Fig. S2** Correlations matrix of all image statistics that were *in consideration* for use as predictors in regression. Absolute values of correlation coefficients are shown.

## **Fixation tendency to critical objects**


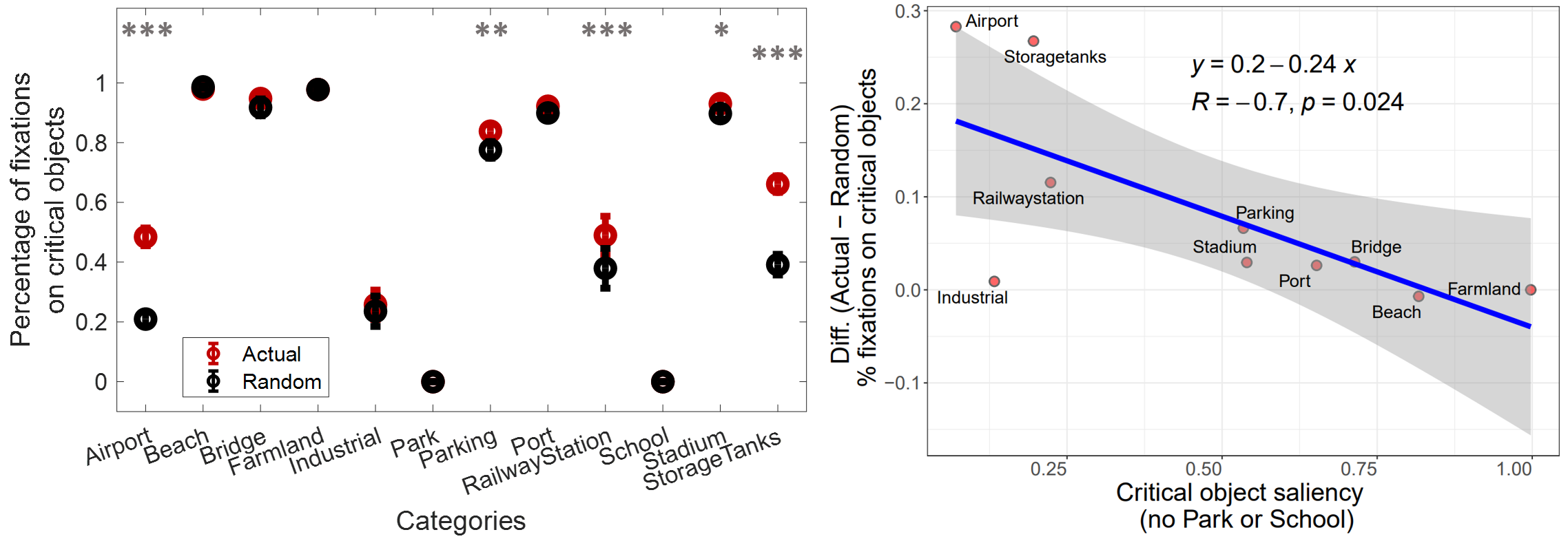
**Fig. S3** For a given image (e.g., *I*), red indicates fixations specific to that image, while black denotes fixations from another randomly chosen image. The two sets of fixations were separately used to calculate the percentage of fixations that fell on critical objects in image *I* (**left**). Fixations falling within or at a distance of less than 0.5 dva from an annotated boundary were considered to land on the object defined by the polygon or rectangle. This 0.5 dva was used to compensate the potential eye tracking error (Wu & Wolfe, 2022). The correlation between Critical Object Saliency (COS) values and the differences in the percentage of on-critical-object fixations between the two sets of fixations (**right**). Asterisks denote significant differences in results between actual and random fixations. Error bars indicate SEM across 20 images per category (*** *p* < .001, ** *p* < .01, * *p* < .05).

## **Response time and categorization difficulty**


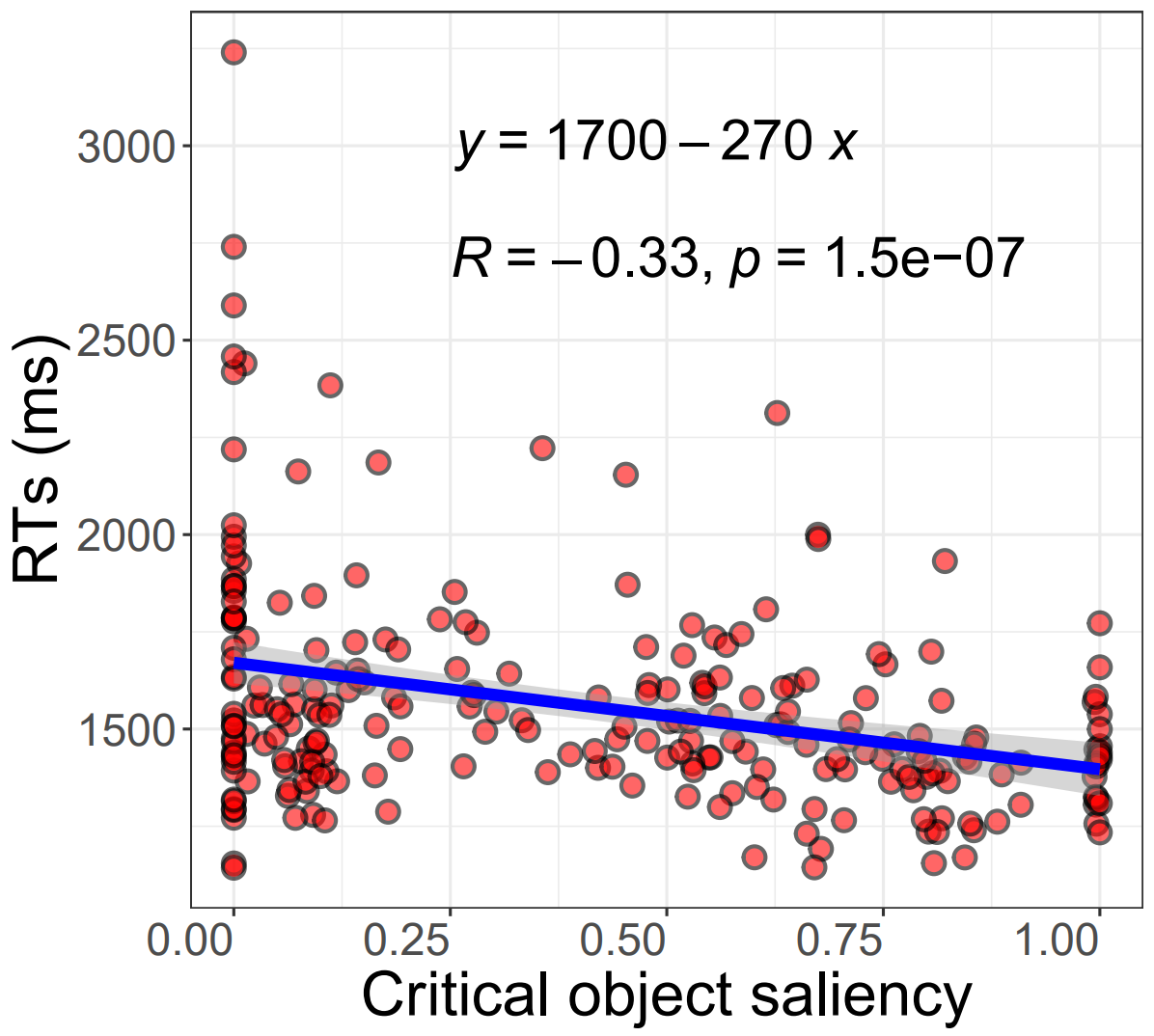

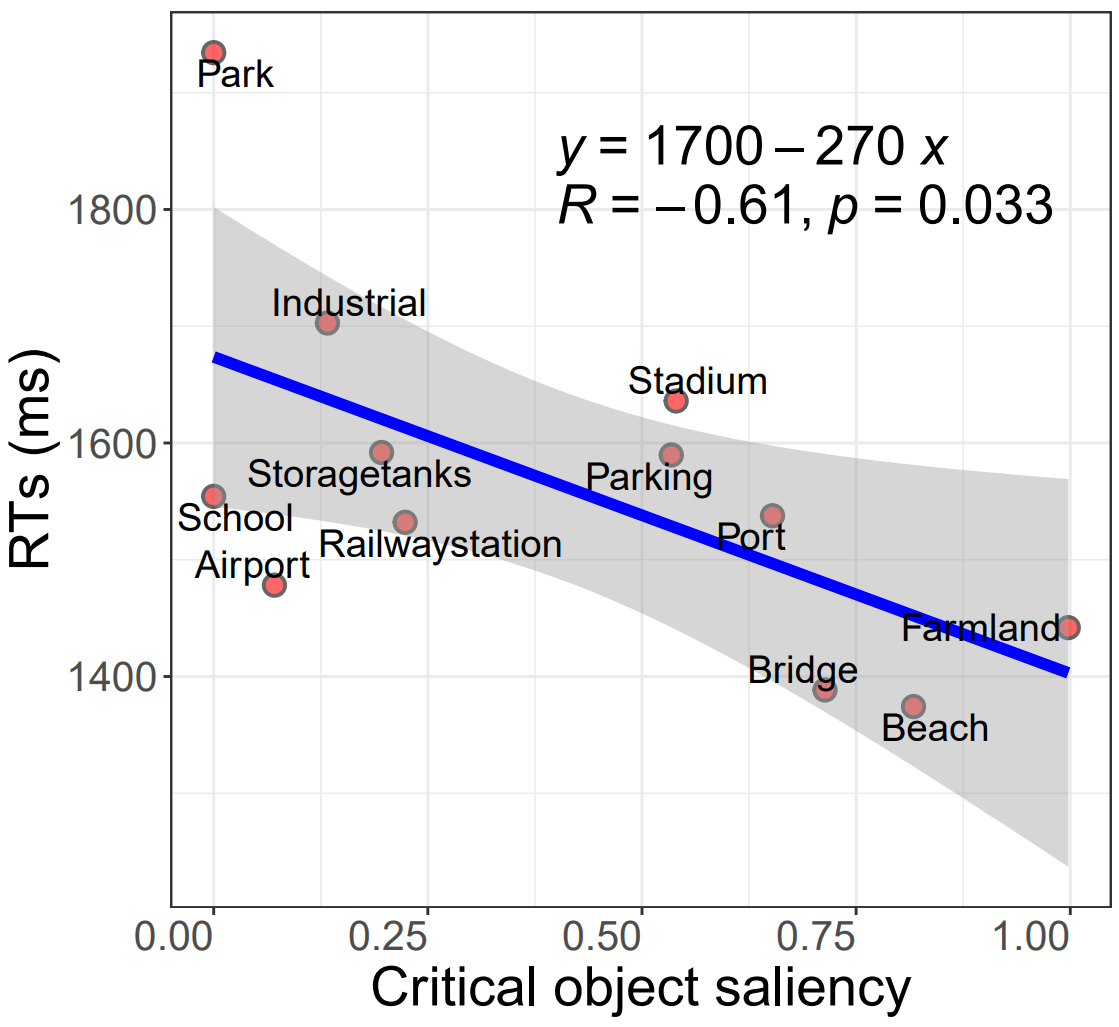


**Fig. S4** Correlations between response times and critical object saliency at the image (**left**) and scene category level (**right**). The response time was from the onset of the 4-AFC task to the moment a decision was made. Participants were instructed to prioritize accuracy over speed. Although the recorded RTs may not represent participants’ fastest performances and were susceptible to noises, there is unlikely a speed-accuracy trade-off. When data from Park and School scenes was excluded, *p* = 2e-05 (image-level) and *p* = .08 (scene category-level).

**Fig. S5** Self-reported scene categorization difficulty for each category. Circles and bars indicate the Mean values and Standard Deviations across 20 participants. After finishing the experiment, a questionnaire was given to participants to report the categorization difficulty for each scene category, using a five-point Likert scale, where 1 meant “pretty easy” while 5 meant “pretty difficult”. Observers did not receive explicit instruction to use this scale to produce five groups of stimuli. Participants used the scale as they saw fit, producing 1 - 5 groups.

**References**

Belsley, D. A., Kuh, E., & Welsch, R. E. (2005). *Regression diagnostics: Identifying influential data and sources of collinearity*. John Wiley & Sons.

Fox, J., & Weisberg, S. (2011). *An R companion to applied regression*. Sage publications.

O’Brien, R. M. (2007). A caution regarding rules of thumb for variance inflation factors. *Quality & quantity*, *41*, 673-690.

Ozturk, I., & Ullah, S. (2022). Does digital financial inclusion matter for economic growth and environmental sustainability in OBRI economies? An empirical analysis. *Resources, Conservation and Recycling*, *185*, 106489.

Wu, C. C., & Wolfe, J. M. (2022). The Functional Visual Field(s) in simple visual search. *Vision research*, *190*, 107965.

Sun, M., Deng, Y., Li, M., Jiang, H., Huang, H., Liao, W., Liu, Y., Yang, J., & Li, Y. (2020). Extraction and Analysis of Blue Steel Roofs Information Based on CNN Using Gaofen-2 Imageries. *Sensors (Basel, Switzerland)*, *20*(16), 4655.
